# Genotyping and phylogenetic location of one clinical isolate of *Bacillus anthracis* isolated from a human in Russia

**DOI:** 10.1186/s12866-019-1542-3

**Published:** 2019-07-17

**Authors:** Sergey V. Pisarenko, Eugene I. Eremenko, Alla G. Ryazanova, Dmitry A. Kovalev, Nina P. Buravtseva, Lyudmila Yu. Aksenova, Anna Yu. Evchenko, Olga V. Semenova, Olga V. Bobrisheva, Irina V. Kuznetsova, Tatyana M. Golovinskaya, Dmitriy K. Tchmerenko, Alexander N. Kulichenko, Vitaliy Yu. Morozov

**Affiliations:** 1 Stavropol Research Anti-Plague Institute, 13-15 Sovetskaya Str, Stavropol, 355035 Russia; 2 Stavropol State Agrarian University, Stavropol, 355017 Russian Federation

**Keywords:** *Bacillus anthracis*, *Russia*, *Whole genome sequencing (WGS)*, Whole-genome single-nucleotide-polymorphism analysis (wgSNP), *Comparative genomics*, *Single nucleotide polymorphism (SNP)*, *Multiple locus variable number of tandem repeat analysis (VNTR*, *MLVA)*

## Abstract

**Background:**

Anthrax is a zoonotic disease caused by the Gram-positive bacterium *Bacillus anthracis*. In Russia, there are more than 35 thousand anthrax stationary unfavourable sites. At the same time, there is very little published information about the isolates of *B. anthracis* from the territory of Russia. In this study, we report the use of whole genome sequencing (WGS) and bioinformatics analysis to characterize *B. anthracis* 81/1 strain isolated in Russia in 1969 from a person during an outbreak of the disease in the Stavropol region.

**Results:**

We used 232 *B. anthracis* genomes, which are currently available in the GenBank database, to determine the place of the Russian isolate in the global phylogeny of *B. anthracis*. The studied strain was characterized by PCR-based genetic methods, such as Multiple-Locus Variable-Number Tandem Repeat Analysis (MLVA), canonical single nucleotide polymorphisms (canSNP), as well as the method of full-genomic analysis of nucleotide polymorphisms (wgSNP). The results indicate that the Russian *B. anthracis* 81/1 strain belongs to Trans-Eurasion (TEA) group, the most representative in the world.

**Conclusions:**

In this study, the full genomic sequence of virulent *B. anthracis* strain from Russia was characterized for the first time. As a result of complex phylogenetic analysis, the place of this isolate was determined in the global phylogenetic structure of the *B. anthracis* population, expanding our knowledge of anthrax phylogeography in Russia.

**Electronic supplementary material:**

The online version of this article (10.1186/s12866-019-1542-3) contains supplementary material, which is available to authorized users.

## Background

Anthrax is a particularly dangerous zoonotic disease, the causative agent of which is an aerobic Gram-positive spore-forming microorganism *Bacillus anthracis*. The relevance of the causative agent of anthrax increased significantly after its use as an agent of bioterrorism in 2001 [1]. In the past, the governments of several countries, including the United States, the United Kingdom and the former Soviet Union, used *B. anthracis* to develop and build biological weapons [2].

In addition to the danger posed by the anthrax pathogen as an agent of bioterrorism, there are other pressing problems associated with anthrax in wildlife, livestock and humans [3]. Wild and domestic herbivorous mammals that ingest or inhale the spores of the pathogen during grazing are most often exposed to anthrax. Human infection usually occurs through contact with diseased animals and raw materials of animal origin or consumption of infected products. In recent years, a new form of anthrax infection, called injectional anthrax, caused by heroin contaminated with anthrax spore, emerged [4]. Annually, between 20.000 and 100.000 cases of anthrax are reported worldwide [5]. The most endemic regions are the countries of sub-Sahara Africa, Central Asia, the Middle East and South America [6, 7]. Nowadays there are more than 35 thousand so-called anthrax stationary unfavourable sites in Russia. According to Cherkasskiy, Russian definition of stationary unfavourable site (place, point or spot) is: rural inhabited place (settlement or village) or part of area, etc. where one or more incidents of anthrax in animals and/or humans was registered independent of the scarcity of their occurrence [8, 9]. The largest number of cases of the disease is recorded in the administrative territories of Siberia and southern Russia [10].

*B. anthracis* 81/1 strain was isolated from a cutaneous lesion of a person infected with anthrax while slaughtering of an ill cow during an outbreak of anthrax in livestock at Rasshevatskoye village in Novoalexandrovskiy district of Stavropol Territory in 1969. Anthrax in this stationary unfavourable site was registered in human and livestock twelve times since 1935.

The development of next generation sequencing (NGS) technologies has allowed significant progress in studying the population structure and evolution of the *B. anthracis* isolates. In contrast to taxonomic informative or canonical SNP-based approaches, sequencing of entire genomes is a reliable method for resolving intraspecific relationships. CanSNP-typing and comparison of the complete genomes of several strains is now becoming the reference method of *B. anthracis* genotyping [11, 12].

There is very little published information in international databases about the *B. anthracis* strains isolated on the territory of Russia. In the most comprehensive GenBank database, where the genome-wide sequences of more than 200 *B.anthracis* strains from different geographical regions of the world are stored, the genomes of isolates from Russia are represented by only three *B. anthracis* Tsiankovskii-I, *B. anthracis* STI-1 and *B. anthracis* 55-VNIIVViM vaccine strains. The only genome of the virulent strain from Russia described in the international scientific literature is the genome of the strain that caused the outbreak of anthrax in 1979 in Sverdlovsk, data on which was obtained by analyzing DNA extracted from two samples of autopsy material [13–16].

Current understanding of the genetic structure of the global population of the anthrax pathogen must be supplemented by clarifying the place of other strains of Russian origin, including virulent ones. In this study, we describe the complete sequence of the genome of *B. anthracis* 81/1, isolated from a person during an anthrax outbreak in southern Russia. We used the canSNP and MLVA genotyping methods, as well as whole-genome SNP analysis (wgSNP) to determine the phylogenetic location of the Russian isolate in the global population of *B. anthracis*.

## Results

### General genome characteristics of *B. anthracis* 81/1 isolate

The total length of the genomic sequence for *B. anthracis* 81/1 was 98.88% (5.442.544 bp) compared to the reference genome Ames Ancestor. GC-content of 81/1 was 35.09%, which is fairly close to that of the reference Ames Ancestor strain (35.24%). Annotation of the genome was performed using the NCBI Prokaryotic Genome Annotation Pipeline. A total of 5.798 genes encoding proteins were predicted; for 5.157 genes (88,9% of the total number of predicted genes) predictable functions were determined. This Whole Genome Shotgun project has been deposited at GenBank under the accession RQWM00000000. The version described in this paper is version RQWM01000000.

### PCR-based genotyping of *B. anthracis* 81/1 isolate

**Fig. 1:**
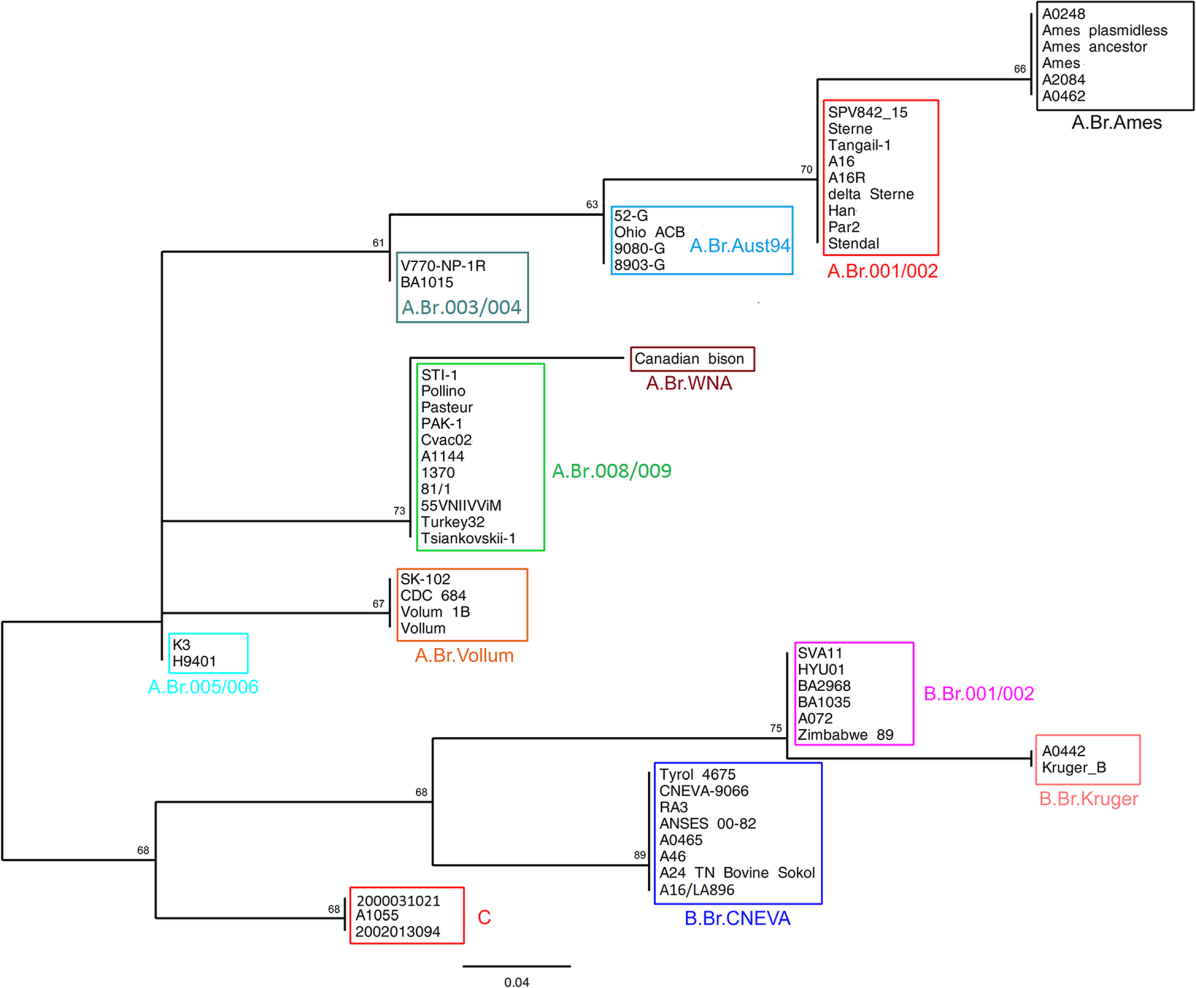
Phylogeny of the major canSNP groups of *B. anthracis*

To identify possible close relatives of the Russian isolate, we performed a canSNP analysis and MLVA-31 of isolate *B. anthracis* 81/1 as described in Methods. We selected for comparison 23 genomes for which we were able to get MLVA-31 profiles in the MLVAbank for microbes genotyping database [17], as well as 35 genomes from the GenBank database, for which it was possible to conduct MLVA-31 and canSNP analysis in silico, using an online resource MicrobesGenotyping [18]. Genotyping based on canSNP analysis distributes the studied strains into 11 canSNP groups (Fig. 1). The phylogenetic relationships between 58 *B. anthracis* strains were inferred by the Tamura 3-parameter model. It is noted that 39 strains belong to the main genetic line A, in which the most representative is subgroup A.Br.008/009 (11 strains). Strain *B. anthracis* 81/1 is included in this subgroup, along with three other strains of Russian origin (STI-1, Tsiankovskii-1 and 55VNIIVViM). In the subgroup A.Br.008/009 there are also strains isolated in Italy, France, Turkey, Pakistan, China and Argentina. This subgroup is dominant in Europe and accounts for a significant share of Asian isolates. Table S1 (see Additional file 1) lists canSNP profiles for 58 strains of *B. anthracis*.

**Fig. 2:**
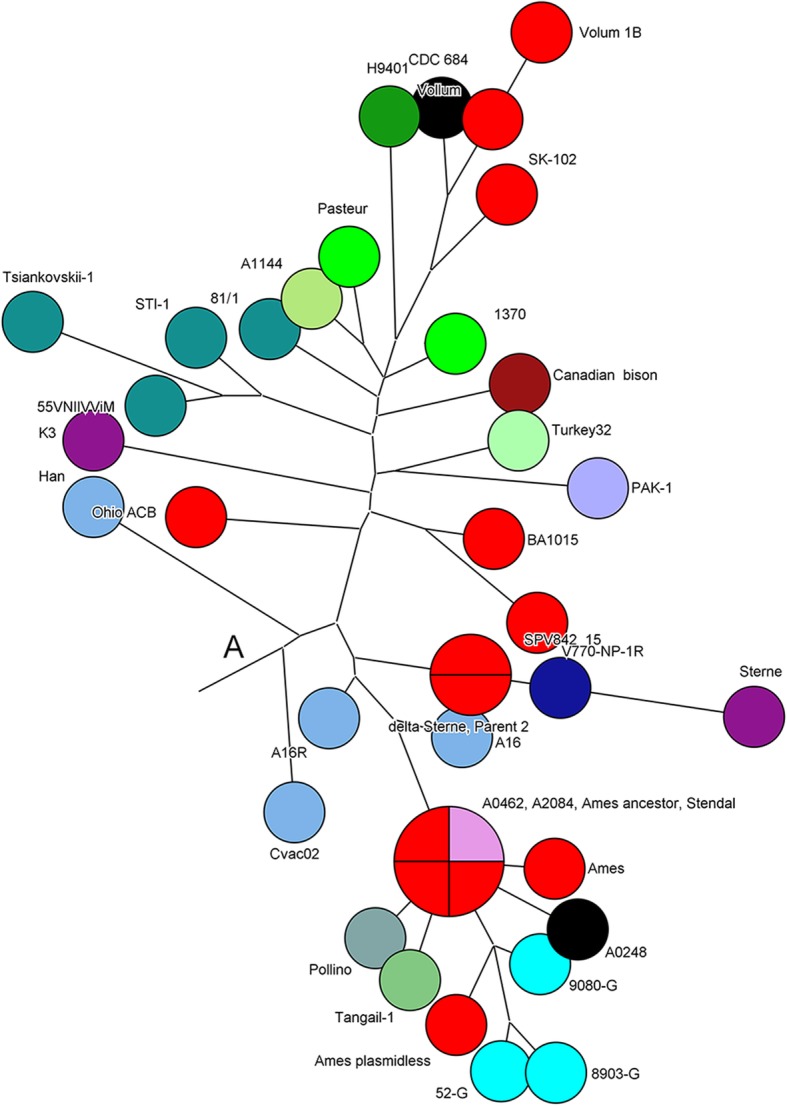
Сlose-up of describing the line A of MLVA31 minimum spanning tree for 58 *B. anthracis* strains

We used data on the number of repeats in the 31 VNTR locus for phylogenetic reconstruction. Thus, we were able to estimate the MLVA similarity between the Russian strain and other strains of different geographical origin. Visualization of the minimum spanning tree based on the number of tandem repeats using the categorical distance coefficient, is shown in Fig. S1 (see Additional file 2). In Table S2 (see Additional file 3) we give MLVA-31 profiles for *B. anthracis* 81/1 strain and 57 other strains *B. anthracis*. MLVA-31 identifies 54 different genotypes in 58 *B. anthracis* strains. There are three main genetic lines A, B and C, but clustering in their structure is more detailed and does not fully coincide with the expected results of canSNP-analysis. A total of 14 clusters are observed, including the main line C, in which three strains have a different MLVA-31 genotype (Fig. 2). Line A is divided into eight clusters and three separate branches for strains Sterne, H9401 and K3. Strains Vollum, Vollum 1B, SK-102 and CDC 684 form a single cluster, which corresponds to belonging to the canSNP subgroup A.Br.Vollum (Fig. 2). Strains belonging to the same canSNP subgroup A.Br.008/009, MLVA-31 divides into two clusters, with Russian strains STI-1, Tsiankovskii-1and 55VNIIVViM in one of these clusters, and the Russian strain 81/1 - in another, together with strains Pasteur, 1370 (France) and 1144 (Argentina), Turkey32 (Turkey), PAK-1 (Pakistan).

### Global phylogenetic analysis of population *B. anthracis*

**Fig. 3:**
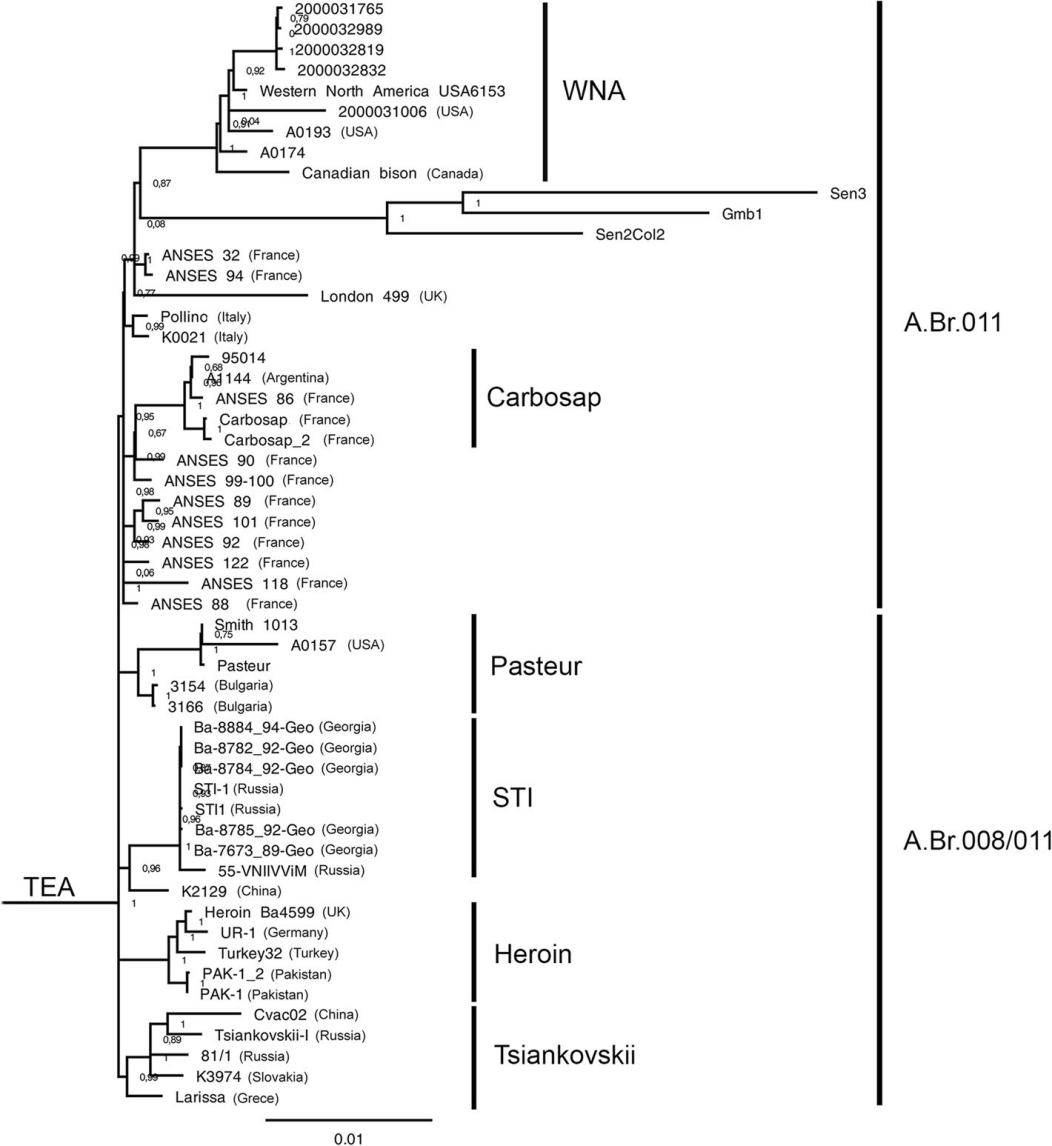
The phylogenetic structure of the TEA group

We used the genomic sequence of the isolate *B. anthracis* 81/1, obtained in this study, and 232 complete genomes *B. anthracis,* available in the GenBank international database, to build a global phylogeny *B. anthracis* based on SNP analysis of complete genomes. Detailed information on the genomes used in the analysis is presented in the Table S3 (see Additional file 4). Genomes of two strains *Bacillus cereus* ATCC14579 (GenBank: GCA_000007825.1) and *Bacillus thuringiensis* 97–27 (GenBank: GCA_000008505.1) were used as an external group. Multiple genome alignment of 235 strains, including external group strains, against the genome of the reference strain *B. anthracis* Ames Ancestor allowed to detect 216.074 SNP. SNP-filtering was perfomed as described in the Methods section. This helped us to get 12.974 SNP totally (see Additional file 5), allowing to differentiate the studied strains. Fig. S2 (see Additional file 6) shows a phylogenetic tree describing the phylogenetic relationships of 233 strains of the species *B. anthracis* used in the analysis. As the result of phylogenetic reconstruction, 49 strains appeared to be of species, different from *B. anthracis*. The number of SNPs found in these strains is many times greater than the number of SNPs found in the rest of the sample strains. These strains included 42 *B. anthracis* strains with names AFS0xxxxx, as well as the strains PFAB2, MCCC 1A02161, N1ZF-2, F34, RIT375, MCCC 1A01412, L19. It is very unlikely that these isolates belong to *B. anthracis;* that is confirmed by the inability to conduct MLVA and SNP typing, as well as to identify *B. anthracis* specific plasmid and chromosomal in silico markers in all of this strains. External group of *B.anthracis* was identified as expected. Taking into account these circumstances, we performed a second round of genome-wide SNP analysis, which used only the genomic sequences of 184 strains identified as *B. anthracis* based on the results of the first round of SNP analysis of complete genomes. Multiple genome alignment of 184 strains to the genome of the reference *B. anthracis* Ames Ancestor strain revealed 9.371 strain-specific SNPs. SNP filtering was performed as described in the section Methods, which allowed to obtain 7.284 SNP totally, which were used for phylogenetic reconstruction. A detailed description of all SNPs used for phylogeny reconstruction can be found in the Table S5 (see Additional file 7). The resulting phylogenetic tree describing the ratios of 184 strains of *B. anthracis*, shown in Fig. S3 [see Additional file 8]. We identified clades of the phylogenetic tree in accordance with the scheme proposed by Sahl et al. [16]. The main genetic line C is formed by five strains forming one cluster. Two clusters corresponding to two canSNP subgroups are observed in the main genetic line B B.Br.CNEVA (9 strains) and B.Br.001/002 (7 strains). The main genetic line A includes 163 strains in six clusters corresponding to subgroups Sterne (49 strains), Aust94 (27 strains), V770 (12 strains), Ancient A (4 strains), TEA Br.008/011 (24 strains), TEA Br.011 (30 strains), Vollum (17 strains). The Russian strain *B. anthracis* 81/1 belongs to cluster TEA Br.008/011, forming together with strains Larisa (Greece), K3974 (Slovakia), Tsiankovskii-1 (Russia), Cvac82 (China) a separate subcluster. The closest to the Russian isolate is the *B. anthracis* K3974 strain isolated in Slovakia in 1995. Other strains of Russian origin STI1, 55VNIIVViM and STI-1 together with strains from Georgia are a part of another subcluster. Figure 3 shows a close-up of a phylogenetic tree describing the cluster TEA structure.

## Discussion

The studied strain was characterized with PCR-based genotyping methods (MLVA andcanSNP) and wgSNP method. The detected discrepancy between the results ofcanSNP and MLVA31 analysis may be explained by the fact that: a) canSNP analysis, unlike MLVA, does not take into account the sequence variability of plasmid DNA; b) VNTR loci of plasmid pXO1aat (vntr19 with repeat unit 3 bp) and of plasmid pXO2at (vntr12 with repeat unit 2 bp) are variable due to the minimum sizes of repeats; c) canSNP analyzes thirteen loci with two possible alleles in comparison to much higher number analyzed with MLVA31 (i.e. 31 loci with the number of alleles from 2 to 13); d) *B.anthracis* SNP-markers have a mutation rate of about 5 × 10–10 mutations/generation, but VNTR-markers of MLVA from < 10–5 to > 10–4 (i.e. SNP-markers are evolutionarily more stable than MLVA-markers and are not susceptible to homoplasy [2]. The high discriminating ability and mutation rate of MLVA31 has a downside, since two isolates from the same outbreak can have a different genotype and can be regarded as having different origins, and susceptibility to homoplasia is fraught with an assessment of strains of different origin with an identical genotype as related. In this regard, it can be concluded that the SNP analysis of the entire genome will ensure both the highest discriminating ability and the high probability of accurate determining the origin of the strain, which are necessary for the epidemiological investigation of the anthrax outbreak.

A full-genomic SNP analysis of 233 *B. anthracis* strains revealed 49 strains that most likely do not belong to this species. The phylogenetic conclusion presented in Fig. S2 suggests that the strains with the names of the species AFS0xxxxx, as well as the strains PFAB2, MCCC 1A02161, N1ZF-2, F34, RIT375, MCCC 1A01412, L19 were mistakenly referred to *B. anthracis*. Another argument in favor of this statement is the number of SNPs found in these strains compared to strains assigned to the main genetic lines A, B, and C. For example, for strains named AFS0xxxxx, the number of SNPs is from 494 to 3.412, for strains PFAB2, MCCC 1A02161, N1ZF-2, F34, RIT375, MCCC 1A01412, L19, the number of detected SNPs is in the range 652–1.262. In our opinion, this number of SNPs is too large for strains belonging to the same species. We conducted a comparative analysis of these data with the number of SNPs found in the strains of the external group and the strains relating to the main genetic line C. For strains *B. cereus* ATCC14579 and *B. thuringiensis* 97–27, the number of SNPs found was 4.943 and 695, respectively, in strains relating to the main genetic line C, the number of SNPs ranges from 68 to 78 compared to the genome of the reference *B. anthracis* Ames Ancestor strain. In our opinion, the number of detected SNPs is highly correlated with the results of similar experiments [19–21].

According to information from the GenBank international database, isolates with species names AFS0xxxxx were isolated from soil and plants in the USA, MCCC 1A02161, N1ZF-2, MCCC 1A01412 and L19 isolates were isolated from sediments in the South China Sea, RIT375 was isolated from Costus igneus stem tissue in Puerto Rico; strains F34 and PFAB2 were isolated from the water of a salt lake in Algeria and a hot spring in India, respectively. With the exception of soil, all these sources of isolation should be attributed to the exotic. We have checked genomes of these strains for specific and validated chromosomal markers as well as plasmid markers for *B. anthracis* including PL3 gene and dhp61 gene [22–24]. None of them show significant matches in any genomes. In addition, these strains are distinguished by a low symmetrical identity relative to *B. anthracis* Ames Ancestor strain and other *B. anthracis* strains (~ 85% and below), whereas for most strains these indicators were not lower than ~ 97%. The clustering of these strains may most likely be explained by erroneous identification. Unfortunately, we were unable to find more complete information about these isolates, which would contain their phenotypic description, canSNP or MLVA-analysis, and could reliably determine the species identity of the isolates.

The global phylogeny of 184 *B. anthracis* strains, built on the basis of the wgSNP analysis, is fully consistent with the accepted idea of the global genetic structure of the anthrax microbe population. Most recent phylogeny based upon this analysis defined three major subclades (A, B and C) with major polytomy named Trans-Eurasion group (TEA) in clade *A. tea* subdivided into TEABr.008/011 (or A.Br.008/011) and TEABr.011 (or A.Br.011) subgroups [16].

The most representative in our study (54 strains) is TEA group (Fig. 3). The *B. anthracis* 81/1 strain belongs to one of the clades of subgroup TEA008/011, namely Tsiankovskii. This clade includes the strains K3974 (Slovakia), Tsiankovskii-I (Russia), Cvac02 (China), and Larissa (Greece). For strains of this clade, from 206 to 271 SNPs were detected in comparison with the genome of the reference strain. In the work of Sahl et al. (2016), the phylogeny of the genome of the strain that caused the outbreak of anthrax in 1979 in Sverdlovsk also places it in the Tsiankovskii clade along with the Cvac02 strain (China). Timofeev et al. [25] also placed two Russian strains isolated in Yakutia into the A.Br.008/011 subgroup. The closest subcluster Heroin consists of five strains, three of which were isolated in Asia and two in Europe. All these strains are associated with anthrax outbreak among drug users in Europe. Earlier, Erin Price and co-authors [26] described the probable route of the Heroin Ba4599 strain, which caused a large outbreak of anthrax among heroin users, to European countries from the Central Asian region. From 227 to 240 SNPs distinguish the strain of this subcluster from the genome of the reference strain. The narrow range of the number of SNPs found in the strains of this clade indicates a high degree of their genetic homogeneity. The third subcluster (STI) includes five strains isolated in Georgia, two vaccine strains from Russia (STI and 55VNIIVViM) and one strain from China. Among these strains, from 214 to 227 SNPs have been found in comparison with the reference, which also indicates a high genetic homogeneity of the strains forming the subcluster. The isolates STI-1 and STI1 are laboratory samples of the acapsular mutant STI-1 isolated by N.N. Ginsburg from the virulent strain of the causative agent of anthrax “Krasnaya Niva”, which became the basis of the Russian live anthrax vaccine STI for animals and people. The genome of the STI-1 strain was sequenced at the Center for Microbial Genetics and Genomics, Northern Arizona University (USA) in 2016. The genome of the STI1 strain was sequenced at the Russian Research Anti-Plague Institute Microbe in 2018. The fourth subcluster (Pasteur) comprises five strains, two of which were isolated in Bulgaria and one in the USA, the origin of two more strains is unknown. For strains of this subcluster, from 202 to 242 SNPs have been found in comparison with the genome of the reference strain.

## Conclusion

As a result of the study, the full genome of virulent *B. anthracis* strain isolated from human in southern Russia was characterized for the first time. Genotyping of the isolate, carried out using the canSNP, MLVA31 and wgSNP methods, made it possible to determine its place in the global population of the anthrax pathogen. The results indicate that the *B. anthracis* 81/1 strain belongs to the subgroup TEA008/11, also known as the Trans-Eurasion group, one of the most representative groups in the world. Our study confirms data on the wide distribution of isolates of this group in Europe, Russia, Kazakhstan, the Caucasus, and in the eastern provinces of China [27]. There is some information about the genomic diversity of *B. anthracis* strains from other Russian regions. It would be advisable to continue the study of the genomes of *B. anthracis* isolates of Russian origin. Such studies would contribute to extending the differentiation of *B. anthracis* strains.

## Methods

### Bacterial strain

The *B. anthracis* 81/1 strain used in this study was isolated from a person diagnosed with anthrax in Stavropol Territory during the outbreak in 1969. The strain was obtained from the State Collection of Pathogenic Microorganisms of Stavropol Research Anti-Plague Institute. The strain was identified using standard biochemical methods in accordance with the requirements of the Russian protocols “Procedure for organizing and conducting laboratory diagnostics of anthrax for laboratories at the territorial, regional and federal levels” (MUK 4.2.2941–11). Cultural, morphological and biochemical properties of the isolate, as well as the results of tests for virulence and antimicrobial susceptibility testing are described in detail in the text application [see Additional file 9]. Spore suspension in sterile distilled water/glycerol in sealed ampules was stored in refrigerator at 4 °C before use.

### Growth of *B. anthracis* and extraction of DNA

Vegetative cells of *B. anthracis* 81/1 strain were cultured on blood agar, then inactivated by incubating in TE buffer (10,0 Tris-HCl [pH 8,0], 1,0 mM EDTA) at 100°С for 20 min. DNA was isolated as previously described [28]. All manipulations with live *B. anthracis* 81/1 strains were performed in a level 3 biosafety laboratory, and the isolated DNA was sterile-filtered (0.1 μm filter pores, Merck Millipore, Germany) before it was taken from the biosafety laboratory level 3. The DNA concentration was quantified using the Qubit dsDNA HS assay kit (Thermo Fisher Scientific, USA) according to the manufacturer’s protocol. DNA preparation was stored at − 20 °C until further use.

### Diagnostic real-time PCR for chromosomal and plasmid markers of *B. anthracis*

For identification of *B. anthracis* by real-time PCR, we used the reagent kit for detecting *Bacillus anthracis* DNA in biological material and environmental objects by the method of PCR with real-time hybridization-fluorescence detection «Amplisens® *Bacillus anthracis*-FRT» (Interlabservice, Russia). Real-time PCR analysis was performed using Rotor-Gene Q (QIAGEN, Germany).

### Analysis of canonical single nucleotide polymorphisms (canSNPs)

For analysis of canonical SNPs, which form 12 groups according to the scheme [29], we used an in-house designed PCR kit with real-time detection and fluorescently labeled LNA probes, a Rotor-Gene Q instrument (QIAGEN, Germany) and the corresponding software Rotor-Gene Q 2.3. Sequences of primers and probes are listed in Table S6 [see Additional file 10]. Phylogenetic relationships between 58 strains of *B. anthracis* were derived in the Mega10 [30] program using the maximum likelihood method based on the Tamura three-parameter model [31]. The bootstrap confidence values were generated using 1,000 permutations.

### Multiple locus variable number of tandem repeats analysis using 31 markers (MLVA-31)

MLVA was performed essentially as described in [32]. Amplification of fragments of 31 marker loci was performed in 7 multiplex PCR. Fragment mixtures were analyzed on a genetic analyzer (ABI 3500, Applied Biosystems, USA) using Genescan 1200 LIZ (Applied Biosystems, USA) as size standards. The data were analysed using the software GeneMapper TM (Applied Biosystems, USA). The MLVA31 cluster analysis was performed using BioNumerics version 7.6 software package (Applied Maths, Belgium). The minimum spanning tree was built based on the number of tandem repeats using the categorical distance coefficient.

### DNA library preparation and whole genome sequencing

We used the methodology previously described by Pisarenko et al. [33]. The preparation of a genomic library with a 400-bp read length was performed using the Ion Xpress Plus Fragment Library Kit reagent kit (Life Technologies, USA) in accordance with the manufacturer’s protocol. DNA library fragments were separated using a ready-to-use commercial kit 2% E-Gel SizeSelect agarose gel (Invitrogen, USA). The finished library of DNA fragments was purified using Agencourt AMPure XP magnetic particles (Beckman Coulter, USA). Library quality and concentration were determined using the Experion™ Automated Electrophoresis System and Experion DNA 1 K Reagents and Supplies and Experion DNA Chips kits (Bio-Rad, USA). Monoclonal amplification on microspheres was performed using Ion PGM Hi-Q View OT2 Kit reagents (Life Technologies, USA) in accordance with the manufacturer’s protocol. Microsphere enrichment was performed using Dynabeads MyOne Streptavidin C1 magnetic particles (Invitrogen, Life Technologies, USA). The effectiveness of the enrichment process was evaluated using the Ion Sphere Quality Control Kit (Life Technologies, USA). Genome sequencing was performed using an Ion Torrent PGM sequencer and Ion 316 Chips Kit V2 chips (Life Technologies, USA).

### Post-sequencing data processing

The quality assessment of the obtained reads was performed using the FastQC version 0.11.3 program [34]. Reads with an average value of quality Q < 20, as well as reads with a length of less than 75 nucleotides were removed in the Trimmomatic version 0.33 program [35]. The genomes were assembled using the Newbler v 3.0 software (Roche, Switzerland), the extent of the genome coverage was more than 80×. Assessment of the quality of genome assembly was performed using the program Quast 5.0 [36], the genomic sequence of *B. anthracis* Ames Ancestor strain (GeneBank: NC_007530.2, NC_007322.2, NC_007323.3) was used as a reference to assess the accuracy and efficiency of the genomic project assembly. Genome annotation was performed using the NCBI Prokaryotic Genome Annotation Pipeline.

### Whole genome SNP analysis

We used the Parsnp tool from the Harvest Suite software for fast multiple alignment of genomic sequences [37]. As input, we used the genome of the strain sequenced by us and the genomic sequences of *B. anthracis* strains from the GenBank public database (see Additional file 4), which were aligned to the chromosomal nucleotide sequence of the reference genome of *B. anthracis* Ames Ancestor (GeneBank: NC_007530.2) using Parsnp (parameters -c -e -u -C 1000). The detected SNPs were extracted to a VCF file, using HarvestTools (version 1.0) from the same software package. To improve the overall quality of the data, SNP positions with a distance of less than 10 bp, as well as positions that carry an unspecified nucleotide (“N”) were removed. The edited file was used as an input file in HarvestTools to compile the FASTA file. Phylogenetic reconstruction was built in Mega10 using the maximum likelihood method (the Maximum Likelihood method) according to the Tamura-Nei model [38]. The bootstrap confidence values were generated using 1.000 permutations.

## Additional Files

Additional file 1 **Table S1**. Data on canSNP 58 strains of *B. anthracis*. (XLSX 23 kb)

Additional file 2 **Figure S1**. Minimum spanning tree of MLVA31 data from 58 *B. anthracis* strains. (TIFF 429 kb)

Additional file 3 **Table S2**. Data on MLVA-31strains of *B. anthracis*. (XLSX 19 kb)

Additional file 4 **Table S3**. Strain metadata. (XLSX 24 kb)

Additional file 5 **Table S4**. Base of sites of nucleotide polymorphisms of 235 *Bacillus* strains. (XLSX 9267 kb)

Additional file 6 **Figure S2**. Phylogenetic clustering based on wgSNP analysis from 235 *Bacillus* strains. (TIF 1822 kb)

Additional file 7 **Table S5**. Base of sites of nucleotide polymorphisms of 184 *B. anthracis* strains. (XLSX 4131 kb)

Additional file 8 **Figure S3**. Phylogenetic clustering based on wgSNP analysis from 184 *B. anthracis* strains. (TIF 2473 kb)

Additional file 9 **Attachment S1**. Biochemical properties and antimicrobial susceptibility testing. (DOCX 12 kb)

Additional file 10 **Table S6**. Sequences of primers and probes for canSNP analysis. (DOCX 14 kb)

## Data Availability

All data generated or analyzed during this study are included in this published article and its supplementary information files.

## Abbreviations

canSNP: Canonical Single Nucleotide Polymorphisms
MLVA: Multiple Loci VNTR Analysis
SNP: Single Nucleotide Polymorphism
TEA: Trans Eurasion group
VNTR: Variable Number Tandem Repeat
WGS: Whole Genome Sequencing
wgSNP: Whole-Genome Single-Nucleotide-Polymorphism
