# Supplementary figures and images for: Genotyping and phylogenetic location of one clinical isolate of Bacillus anthracis isolated from a human in Russia
Source: BMC Microbiol. 2019 Jul 17;19:165. doi: 10.1186/s12866-019-1542-3 (PMC6637652; doi:10.1186/s12866-019-1542-3)

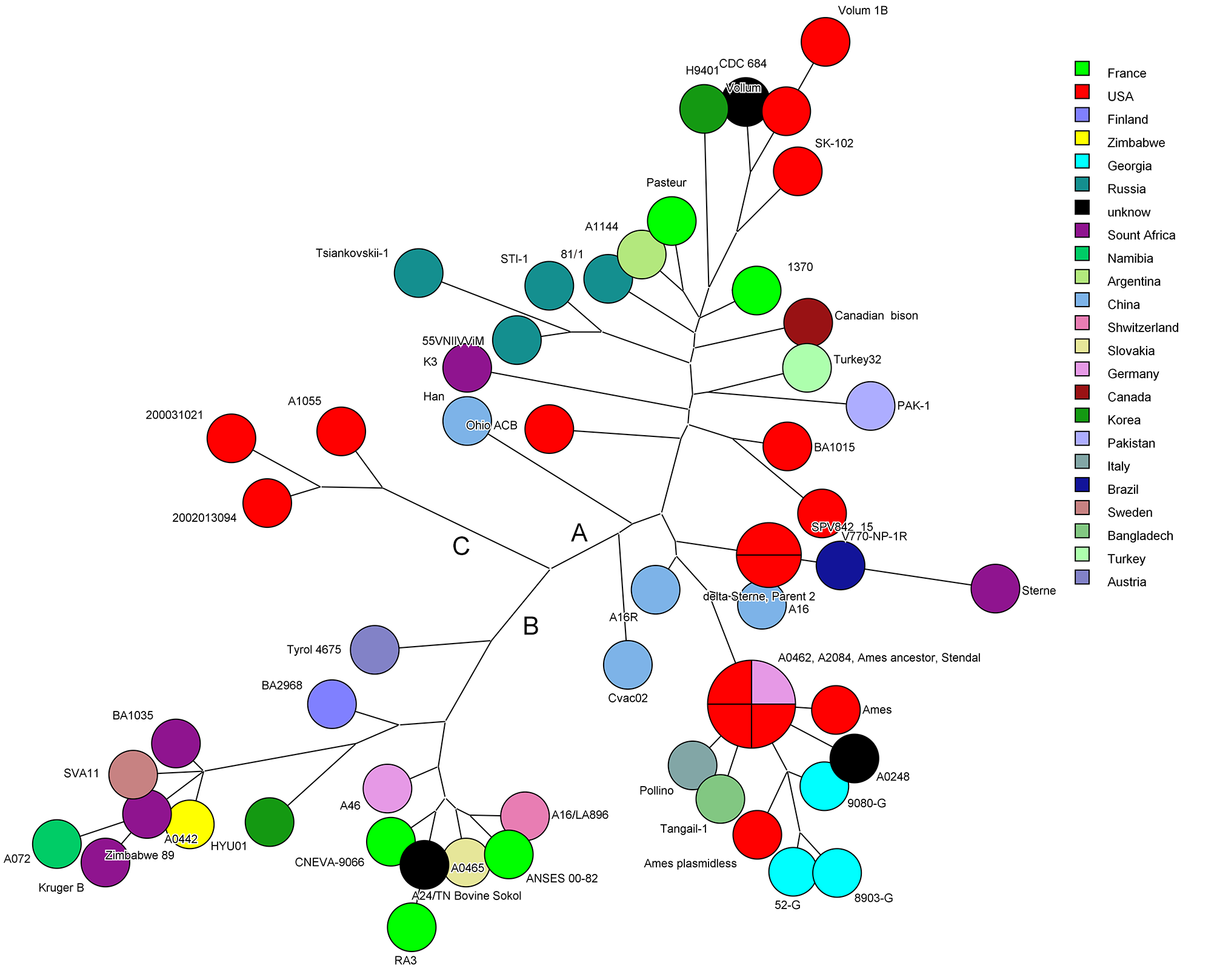

Supplement: Supplementary file 2 — Figure S1. Minimum spanning tree of MLVA31 data from 58 B. anthracis strains. (TIFF 429 kb) [file 12866_2019_1542_MOESM2_ESM.tiff]
