# Supplementary material for: Genotyping and phylogenetic location of one clinical isolate of Bacillus anthracis isolated from a human in Russia
Source: BMC Microbiol. 2019 Jul 17;19:165. doi: 10.1186/s12866-019-1542-3 (PMC6637652; doi:10.1186/s12866-019-1542-3)
Supplement: Supplementary file 9 — Attachment S1. Biochemical properties and antimicrobial susceptibility testing. (DOCX 12 kb) [file 12866_2019_1542_MOESM9_ESM.docx]

**Cultural and morphological properties of the strain**

It is a gram-positive bacillus forming spore, capsule and exotoxins. Non-motile. In solid nutrient media, a typical growth in the R-form is recorded. When cultivating in 1% bicarbonate agar with 10% of inactivated horse serum in an atmosphere with 10–20% CO2, formed large shiny mucous colonies in SM form.

**Biochemical properties of the strain**

Proteolytically active, hydrolyzes casein, gelatine, haemoglobin, albumin and fibrinogen. Penicillin sensitive, alkaline phosphatase, lecitnase and hemolysis negative. Lysed by anthrax bacteriophage "Gamma".

**Antibiotic susceptibility testing and Virulence**

Sensitivity to antibiotics was determined by the disk-diffusion method The strain is resistant to polymyxin and trimethoprim, sensitive to benzyl penicillin, ampicillin, streptomycin, tetracycline, doxycycline, rifampicin, ciprofloxacin. The strain is virulent. In the body of experimental animals forms a capsule and exotoxins. Causes the death of non-linear white mice, guinea pigs, rabbits and sheep 2 to 5 days after subcutaneous challenge.
