# Supplementary material for: Genotyping and phylogenetic location of one clinical isolate of Bacillus anthracis isolated from a human in Russia
Source: BMC Microbiol. 2019 Jul 17;19:165. doi: 10.1186/s12866-019-1542-3 (PMC6637652; doi:10.1186/s12866-019-1542-3)
Supplement: Supplementary file 10 — Table S6. Sequences of primers and probes for canSNP analysis. (DOCX 14 kb) [file 12866_2019_1542_MOESM10_ESM.docx]

Table S6. Sequences of primers and probes for canSNP analysis

| **Can SNP** | **Nucleotide sequence 5’→3’** | |
| --- | --- | --- |
|  | **Primers** | **Probes** |
| A.Br.001 | F:CAAGCGGAACCAAATTTAATCTTT  R:TTCACCGTACGTCATTGTATAATACG | FAM-ACCGAAACTTGAAGTC-BHQ1 |
|  |  | VIC-AACCGAAATTTGAAGTC-BHQ2 |
| A.Br.002 | F:AACGATACCTAAAATCGATAAAG  R:GGCAGAAGGAGCAAGTAATGTT | FAM-CGCCCAGCCTAA-BHQ1 |
|  |  | VIC-CGCCCAACCTAAAC-BHQ2 |
| A.Br.003 | F:GCTACTGTCATTGTATAAAAACCTCCTTT  R:CGCTTGCCAAGCTTTTTTTC | FAM-ACCTCAAGCTTAATTCG-BHQ1 |
|  |  | VIC-CTACCTCAAACTTAATTCGA-BHQ2 |
| A.Br.004 | F:CCGATACCAGTAAACGACGACAT  R:CTGGAATTGGTGGAGCTATGGA | FAM-TGGAATGCCCCTAATC-BHQ1 |
|  |  | VIC-TGGAATGTCCCTAATCC-BHQ2 |
| A.Br.006 | F:CCGGAAATTGCTATTAGAACGAA  R:TCCCAATCTAGCGTTTTTAAGTTCA | FAM-TCGCCTCGTGCAT-BHQ1 |
|  |  | VIC-ATCGCCTAGTGCATG-BHQ2 |
| A.Br.007 | F:TTGGTAACGAGACGATAAACTGAATAA  R:GCCTTGGATTGGCGATTG | FAM-CCATCCTTATATTCAGCT-BHQ1 |
|  |  | VIC-CATCCTTACATTCAGCT-BHQ2 |
| A.Br.008 | F:TTCGCAACTACGCTATACGTTTTAGAT  R:CAAACGGTGAAAAAGTTACAAATATACG | FAM-TTCTTCGCCGCTTG-BHQ1 |
|  |  | VIC-AATTCTTCTCCGCTTGT-BHQ2 |
| A.Br.009 | F:GGCAATCGGCCACTGTTT  R:GGGTTTCTACTGTGTATGTTGTTAATAAAAAG | FAM -GGCTTTGCTTGCATC-BHQ1 |
|  |  | VIC-CGGCTTTACTTGCATC-BHQ2 |
| B.Br.001 | F:TGCATGCTTCTTCTTACAGAGTAGTTAAT  R:CGGTCATAAAAGAAATCGGTACAA | FAM-GATACCTTCTTATCCTCTTC-BHQ1 |
|  |  | VIC-GATACCTTCTTATCTTCTTCT-BHQ1 |
| B.Br.002 | F:TGTTGCACCTTCTGTGTTCGTT  R:GTAGTGGCTTCACCGAATGGA | FAM-ACGTTACTGCTGTTCCT-BHQ1 |
|  |  | VIC-AACGTTACTTCTGTTCCT-BHQ1 |
| B.Br.003 | F:CATTTATTCGCATAGAAGCAGATGA  R:TGTGCCATCAAATAACTCTTTCTCAA | FAM-ACATATCCACTTCACGTT-BHQ1 |
|  |  | VIC-CATATCCGCTTCACGT-BHQ1 |
| B.Br.004 | F:GAAGTTAAGTATCAACCAGCAGAAGAAA  R:CCGCCGCCTTGAGCTT | FAM-TTACTTCTATCATCCCGT-BHQ1 |
|  |  | VIC-TACTTCTACCATCCCGT-BHQ1 |
| A/B.Br.001 | F:GAAGGTCTCCAATTTGGATTTAAAAT  R:CGTGTGAACCTTTCGGTAAATAGTC | FAM-TTTTATTTAGAAGATAGCGC-BHQ1 |
|  |  | VIC-TTTATTTAGGAGATAGCGGC-BHQ1 |

Underlined highlighted LNA bases.
